# 癌症神经科学视角下肺癌演进的神经调控机制与临床治疗挑战

**DOI:** 10.3779/j.issn.1009-3419.2026.106.07

**Published:** 2026-03-20

**Authors:** Na WANG, Bo ZHANG, Lihan SHANG, Xuerui WANG, Xiaoshan WANG, Qiyu FAN, Chaoran WANG, Fanming KONG

**Affiliations:** 300381 天津，天津中医药大学第一附属医院，中医国家临床医学研究中心，天津市中医肿瘤研究所; First Teaching Hospital of Tianjin University of Traditional Chinese Medicine, National Clinical Research Center for Chinese Medicine, Tianjin Cancer Institute of Traditional Chinese Medicine, Tianjin 300381, China

**Keywords:** 肺肿瘤, 癌症神经科学, 神经系统, 神经活性分子, Lung neoplasms, Neuroscience, Neuro-oncology, Neuroactive molecule

## Abstract

肺癌作为全球发病率与死亡率均居首位的恶性肿瘤，其演变进程与神经系统存在高度复杂的双向调控关系。神经系统在肺癌的起源阶段发挥关键调控作用，可通过神经元、神经递质及神经活性分子等核心介导途径，参与肺癌细胞增殖、局部侵袭及远处转移等恶性生物学行为的调控；与此同时，肺癌本身及相关治疗干预措施亦能反向诱导神经系统的结构重塑与功能重编程。癌症神经科学这一新兴交叉学科的崛起，为突破肺癌治疗瓶颈提供了全新的研究视角，但同时也面临诸多亟待解决的理论与临床挑战。本文系统梳理神经系统与肺癌演进的内在关联，重点剖析局部治疗、化疗、免疫治疗及靶向治疗等临床策略对神经系统的潜在影响及其分子机制，进一步明确该领域的研究机遇与核心挑战，旨在为优化肺癌精准治疗体系、提升临床治疗效能提供理论依据与实践参考。

## Abstract (en)

Lung cancer is the leading cause of cancer morbidity and mortality worldwide. A highly complex bidirectional regulatory relationship exists between lung cancer progression and the nervous system. The nervous system is believed to play a pivotal regulatory role in the genesis of lung cancer. It is involved in regulating malignant biological behaviors such as proliferation, local invasion, and distant metastasis of lung cancer cells via core mediating pathways involving neurons, neurotransmitters, and neuroactive molecules. At the same time, lung cancer itself and related therapeutic interventions are capable of inducing reverse structural and functional reprogramming within the nervous system. The emergence of cancer neuroscience as a novel interdisciplinary field offers a new perspective to overcome the current bottlenecks in lung cancer treatment, yet it also faces numerous theoretical and clinical challenges that urgently require resolution. This article systematically reviews the intricate relationship between the nervous system and lung cancer progression, focusing on the potential effects and molecular mechanisms of clinical strategies including local therapies, chemotherapy, immunotherapy, and targeted therapy on the nervous system, with the aim of clarifying research opportunities and key challenges in this field and providing a theoretical foundation and practical reference for optimizing the precision treatment system of lung cancer and improving clinical efficacy.

“癌症神经科学（cancer neuroscience）”这一新兴交叉领域的迅速发展，肿瘤与神经系统之间的复杂双向调控网络正被逐步揭示，成为当前肿瘤生物学研究的前沿焦点。近年来，研究^[1]^证据表明，神经系统在肿瘤的多阶段演进过程中发挥着不可或缺的驱动与调控作用，其影响范围涵盖肿瘤发生的早期启动、原发灶的局部侵袭乃至远处器官的转移定植等关键环节。神经系统作为维系机体内环境稳态的核心调控枢纽，通过电活动整合、神经递质介导的突触通信以及细胞因子驱动的旁分泌信号，实现对多器官系统的精细化动态调节。在肿瘤进展过程中，不同维度的神经参与现象广泛存在，如肿瘤细胞主动诱导神经纤维向肿瘤微环境浸润，以及原发肿瘤及其治疗干预所引发的一系列神经毒性症状^[2]^。这些病理变化不仅凸显了肿瘤-神经互作的高度复杂性，更与患者不良预后密切关联，并显著增加临床诊断与治疗的挑战。癌症神经科学旨在系统阐明神经系统与肿瘤之间的双向信号调控机制。肿瘤细胞能够调节神经内分泌（neuroendocrine, NE）相关信号途径，从结构与功能层面对神经系统进行重塑，进而形成病理性正反馈环路，导致神经损伤并进一步促进肿瘤进展。

随着该领域研究的不断深入，癌症神经科学将为解析肿瘤发生与进展的关键生物学机制、识别具转化潜力的新型治疗靶点以及推动难治性肿瘤治疗策略的革新提供重要契机。本研究围绕肺癌与神经系统相互作用这一核心科学问题展开系统论述，结合临床常用的局部治疗、化疗、免疫治疗及靶向治疗等，评述现有治疗策略对神经系统结构完整性与功能稳态的影响，进一步明确治疗相关神经毒性的临床表型、发生机制及其潜在意义，总结了癌症神经科学领域的最新进展与未来机遇，并指出当前研究面临的关键挑战。

## 1 神经系统调控肺癌演进的机制

### 1.1 神经系统参与肺癌起源

肺癌主要分为小细胞肺癌（small cell lung cancer, SCLC）与非小细胞肺癌（non-small cell lung cancer, NSCLC）两大亚型。二者的起源特征不同，与神经系统的调控关联亦存在显著差异，神经系统在其起源阶段已发挥核心调控作用。SCLC源自NE细胞，具有增殖速度快、早期广泛转移、对常规治疗高度敏感而易复发等生物学特征，导致其总体预后极差，5年生存率不足10%^[3]^。研究^[4]^证实，SCLC中NE细胞与non-NE细胞构成的混合细胞群，其转移能力显著强于单纯NE细胞。这种细胞异质性赋予SCLC转移潜能的核心路径为，non-NE细胞分泌成纤维细胞生长因子2（fibroblast growth factor 2, FGF2），进而诱导NE细胞中促转移转录因子多瘤病毒增强子激活因子3（polyomavirus enhancer activator 3, PEA3）的表达上调^[5]^。另一方面，神经系统作为 SCLC进展的外在调控系统，可通过多种神经因子，靶向调控肺癌细胞中与轴突形成及神经元迁移相关通路的基因程序表达，促使SCLC细胞以轴突样突起的形式生长，进而获得更强的侵袭与转移能力^[6]^。此外，神经纤维可与SCLC细胞表达的β2-肾上腺素能受体（beta-2 adrenergic receptor, ADRB2）相互作用，最终促进SCLC细胞的增殖^[7]^。综上，SCLC的NE起源属性与神经系统的外在调控作用形成协同效应，共同驱动了其高度侵袭与转移的生物学特征。

NSCLC并非源自NE细胞，但其在侵袭扩散尤其是脑转移发展过程中，与神经系统之间呈现出高度紧密的病理性互作关系。既往研究^[8]^证实，NSCLC在接受化疗、免疫治疗或靶向治疗等多重临床干预压力后，可能发生NE转化，部分病例进一步演变为SCLC转化。此过程中NSCLC细胞会获得与SCLC相似的生物学特性，同时表现出更强的治疗抵抗性和侵袭性，是晚期NSCLC治疗失败的重要机制。此外，MYC原癌基因（MYC proto-oncogene, MYC）作为核心转录调控因子，可通过调节表观遗传调控元件增强细胞可塑性与类干性状态，在多种肿瘤类型中促进细胞谱系重塑，包括SCLC在内的多种NE肿瘤普遍呈现MYC扩增，进一步支持其在NE转化早期阶段中的关键驱动作用^[9]^。综上，这些分子与信号通路的异常改变共同参与了NSCLC向NE表型的转化过程，并驱动其进一步演变为SCLC（图1）。

**图1:**
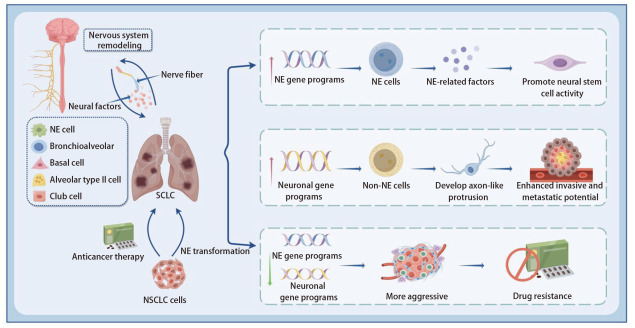
神经系统调控与SCLC起源及NSCLC神经内分泌转化的相关机制

肺腺癌（lung adenocarcinoma, LUAD）与肺鳞癌（lung squamous cell carcinoma, LUSC）作为NSCLC的主要亚型，与神经系统的相互作用模式呈现亚型特异性异质性，成为其起源后恶性表型分化的重要调控因素，主要体现在神经依赖程度、神经递质受体表达谱及谱系可塑性等方面^[10]^。LUAD与神经系统的互作以神经调控促进肿瘤进展为主要特征。转录组学分析显示，LUAD组织中ADRB2及烟碱型乙酰胆碱受体（nicotinic acetylcholine receptor, nAChR）表达显著上调，且与不良预后密切关联；交感神经兴奋可通过β-肾上腺素能受体/环磷酸腺苷/蛋白激酶A（beta-adrenergic receptor/cyclic adenosine monophosphate/protein kinase A, β-AR/cAMP/PKA）信号通路激活丝裂原活化蛋白激酶（mitogen-activated protein kinase, MAPK）及磷脂酰肌醇3-激酶/蛋白激酶B（phosphatidylinositol 3-kinase/protein kinase B, PI3K/AKT）等下游效应轴，促进肿瘤细胞增殖与血管生成。多变量Cox回归分析^[11]^进一步证实，在校正年龄、分期及驱动基因状态等混杂因素后，ADRB2高表达仍为独立不良预后因素。相比之下，LUSC与神经系统的交互更侧重于局部神经侵袭及炎症微环境调控。病理学研究^[12]^表明，LUSC中神经周围浸润发生率高于LUAD，且与局部复发风险增加显著关联；差异表达分析显示，神经生长因子、脑源性神经营养因子及其受体原肌球蛋白受体激酶（tropomyosin receptor kinase, TRK）A/B在LUSC中表达升高，可能通过神经营养因子介导的轴突发生促进神经纤维向肿瘤组织生长，为肿瘤的局部恶性进展奠定微环境基础。

### 1.2 神经系统促进肺癌侵袭与转移

除参与肺癌的发生起源外，神经系统还可通过精密的神经网络调控及多样的神经元源性分子介导，实现对肺癌细胞侵袭、转移等恶性生物学行为的精准调控。反之，肺癌细胞亦可通过主动重塑神经系统结构与功能，形成促进自身侵袭转移的病理性正反馈环路。肺癌细胞可通过调控多种NE相关因子影响肿瘤微环境中的神经网络重塑，为自身侵袭转移构建有利的神经微环境。在SCLC中高表达的Notch配体Delta样配体3（delta-like ligand 3, DLL3）已被证实能够促进神经干细胞活化并增强神经发生过程，推动肿瘤微环境内神经纤维的新生^[13]^。同时，肺癌细胞可分泌神经生长因子、脑源性神经营养因子等神经营养因子，诱导交感神经、副交感神经及感觉神经等多种神经纤维向肿瘤组织定向生长，形成复杂的肿瘤相关神经支配网络。

更关键的是，肺癌细胞可与侵入肿瘤组织的神经纤维形成类似突触的神经-肿瘤连接结构，通过电信号或神经递质介导的化学信号传递，实现肿瘤细胞与神经系统之间的功能性交互，这是神经系统促进肺癌侵袭转移的核心分子机制，可直接驱动肿瘤细胞的局部侵袭、远处迁移及转移定植^[14]^。除直接的神经信号调控外，肺癌细胞还可通过重塑神经胶质细胞微环境进一步增强神经-肿瘤互作。例如肿瘤细胞能够募集并激活分泌神经营养因子的星形胶质细胞，从而促进肺癌细胞增殖并形成神经-肿瘤正反馈调控环路^[15]^。与此同时，机体针对肺癌抗原产生的自身抗体亦可能对神经系统产生影响。部分肿瘤相关自身抗体可作用于神经元表面受体并改变神经元兴奋性，进而诱导神经系统损伤和功能障碍。

### 1.3 神经-免疫调控与免疫逃逸

神经系统通过神经递质、神经营养因子及NE信号轴构成复杂的调控网络，其对肺癌侵袭转移的调控并非单一信号通路的独立作用，而是通过深度整合神经-免疫调控网络，系统性重塑肿瘤免疫微环境、诱导肿瘤免疫逃逸，进而间接为肺癌的恶性进展创造有利条件，同时该调控网络也是影响肺癌免疫治疗疗效的核心因素之一。作为维持机体稳态的重要调节系统，神经系统可通过神经-免疫-内分泌信号网络多维度影响肿瘤细胞生物学行为，其与免疫系统之间存在高度复杂的双向调控关系，其中神经递质、神经肽及NE信号可直接参与抗肿瘤免疫反应的调控^[16]^。在肺癌微环境中，交感神经兴奋后释放的去甲肾上腺素可特异性激活β-AR信号通路，进而诱导肿瘤细胞及肿瘤相关免疫细胞分泌大量免疫抑制性细胞因子，直接削弱CD8^+^ T细胞的细胞毒杀伤功能，显著抑制机体固有及适应性抗肿瘤免疫反应^[10]^。同时，P物质、降钙素基因相关肽及γ-氨基丁酸等多种神经肽，可通过精准调控肿瘤相关巨噬细胞、树突状细胞、调节性T细胞等免疫细胞的活化、分化及功能状态，打破肿瘤微环境中免疫效应细胞与免疫抑制细胞之间的动态平衡，进一步加剧免疫抑制微环境的形成，为肺癌细胞的侵袭转移提供免疫屏障。

### 1.4 神经微环境与软脑膜转移

软脑膜转移（leptomeningeal metastasis, LM）是晚期肺癌，尤其是表皮生长因子受体（epidermal growth factor receptor, EGFR）突变阳性NSCLC及SCLC患者中预后极差的中枢神经系统（central nervous system, CNS）并发症。随着第三代EGFR-酪氨酸激酶抑制剂（EGFR-tyrosine kinase inhibitors, EGFR-TKIs）显著延长EGFR突变阳性NSCLC患者的生存期，其LM检出率呈上升趋势，该病早期诊断困难、治疗反应有限的临床特征已成为肺癌中枢转移管理的核心难点^[17]^。流行病学资料^[18]^显示，EGFR突变阳性NSCLC患者LM发生率为9%-15%，SCLC患者LM发生率更高，可达15%-30%，两类患者发生LM后的中位生存期多不足6个月，远低于未发生LM的晚期肺癌患者。癌症神经科学视角认为，LM并非单纯解剖学播散，而是其对脑膜-脑脊液神经生态位的主动适应性重塑结果。在代谢适应层面，肿瘤细胞通过增强脂肪酸氧化及氧化磷酸化重编程，适配脑脊液低营养微环境，并依托EGFR信号通路维持存活优势；在神经胶质细胞调控层面，肿瘤细胞诱导星形胶质细胞分泌白细胞介素-6（interleukin-6, IL-6）、C-X-C基序趋化因子配体12（C-X-C motif chemokine ligand 12, CXCL12）等细胞因子，通过激活信号转导及转录激活因子3（signal transducer and activator of transcription 3, STAT3）/PI3K/AKT下游信号轴强化恶性表型，同时驱动小胶质细胞向免疫抑制表型极化，构建免疫逃逸微环境；在屏障重塑层面，炎症反应介导紧密连接蛋白表达下调，同时上调血管细胞黏附分子-1（vascular cell adhesion molecule-1, VCAM-1）、细胞间黏附分子-1（intercellular adhesion molecule-1, ICAM-1）等黏附分子，促进肿瘤细胞外渗与脑膜定植；在趋化迁移层面，神经营养因子及β-肾上腺素能信号通路的异常激活，为肿瘤细胞提供定向趋化与迁移优势，加速LM进展^[19,20]^。目前LM的诊断仍依赖增强磁共振成像（magnetic resonance imaging, MRI）与脑脊液细胞学检查，整体灵敏度与特异度有限。脑脊液循环肿瘤DNA及炎症因子谱等液体活检技术，有望显著提升早期诊断效能与分子分型精准度。治疗以血脑屏障（blood-brain barrier, BBB）TKIs、鞘内化疗及局部放疗为核心策略，但疗效受BBB穿透受限、肿瘤分子异质性及治疗相关神经毒性等多重因素制约。未来研究应聚焦神经胶质-肿瘤互作信号轴、神经递质调控及高效跨BBB递药系统，构建多维度精准诊疗与综合干预新模式。

### 1.5 神经-肿瘤互作研究的数字化解析与调控新策略

随着癌症神经科学研究的不断深入，如何实现神经-肿瘤互作网络的精准解析与可干预调控，已成为该领域的重要发展方向。近年来，人工智能（artificial intelligence, AI）驱动的多模态数据整合技术，为系统揭示肺癌与神经系统之间复杂交互机制提供了关键方法学支撑。

在机制解析层面，基于深度学习的神经影像组学分析能够对脑转移灶的空间分布特征、微环境结构及功能连接改变进行高精度量化，从而客观反映肿瘤对CNS结构与功能重塑的动态过程^[21]^。在此基础上，AI算法进一步整合单细胞转录组学、空间转录组学及影像组学等多维数据，可构建多尺度神经-肿瘤互作网络模型，系统解析神经递质、神经营养因子与免疫炎症信号轴之间的交叉调控机制。这一数据驱动的系统生物学策略，有助于识别驱动肿瘤神经依赖性生长及转移的关键调控节点，从而为靶向干预提供潜在分子靶点^[22]^。在临床转化层面，基于机器学习的预测模型已逐步应用于肺癌治疗相关神经毒性的风险评估。通过整合临床病理特征、治疗暴露模式、影像组学参数及神经功能评估指标，可实现神经毒性的前瞻性风险分层与个体化管理优化。这种多维数据驱动的预测模式，本质上反映了肿瘤治疗对神经系统影响的个体异质性，为精准医学背景下的毒性管理提供了重要决策支持。在干预策略方面，神经调控技术为靶向调节神经-肿瘤互作提供了新的研究路径。既往研究^[23]^表明，自主神经系统，尤其是交感与迷走神经，在肿瘤免疫调控及微环境塑造中发挥关键作用。因此，基于神经电活动调控的干预手段，如迷走神经刺激及闭环神经调控系统，有望通过调节神经-免疫信号轴，间接影响肿瘤进展及治疗相关神经毒性的发生与演变。此外，此类神经调控策略在改善肺癌相关神经系统症状包括癌性神经病理性疼痛、认知功能障碍及情绪异常等方面亦显示出潜在应用价值，从而实现症状干预与机制调控相结合的综合获益。

需要指出的是，当前上述技术在肺癌领域的应用仍主要处于机制探索及早期临床转化阶段，其真实临床效益尚需高质量前瞻性研究进一步验证。未来研究应重点聚焦在神经调控靶点的精准识别及作用特异性评估、神经-免疫-肿瘤网络的动态调控机制及长期干预的安全性及其对肿瘤生物学行为的潜在影响。总体而言，数字化技术与神经调控策略并非独立于癌症神经科学体系之外的附加手段，而是推动神经系统-肺癌相互作用机制解析与精准干预的关键技术支撑。其与传统分子生物学研究的深度融合，有望促进该领域由机制描述向可预测、可干预的临床转化阶段迈进，为肺癌精准治疗体系的优化提供新的理论基础与技术路径。

## 2 肺癌治疗相关神经系统的影响

### 2.1 局部治疗

局部治疗是肺癌综合治疗体系的核心组成部分，以放射治疗和外科治疗为主要手段，介入治疗为重要补充，在原发灶局部控制、寡转移病灶清除及症状性转移灶姑息处理中均具有不可替代的临床价值。与全身治疗相比，局部治疗可通过电离辐射、手术切除及物理或放射性消融等方式实现较高强度的局部杀伤，但由于其作用对象并非完全限于肿瘤细胞，邻近正常神经组织亦可能受到直接或间接损伤。值得注意的是，不同局部治疗方式所致神经系统不良反应并非单一谱系，其在CNS与周围神经系统（peripheral nervous system, PNS）中的受累部位、病理基础、时间演变及临床转归均存在明显异质性。故本节按放射治疗、介入治疗及外科治疗分别阐述其对CNS与PNS的影响及临床管理要点。

#### 2.1.1 放射治疗

放射治疗是肺癌原发灶、纵隔病变及脑转移灶局部控制的一线治疗方式，主要包括调强放射治疗（intensity modulated radiation therapy, IMRT）、立体定向放射外科（stereotactic radiosurgery, SRS）、立体定向放疗（stereotactic radiotherapy, SRT）及全脑放射治疗（whole brain radiotherapy, WBRT）等^[24]^。放疗相关神经毒性的发生基础在于电离辐射对神经元、少突胶质细胞、星形胶质细胞及微血管内皮细胞的直接损伤，并可进一步诱导局部炎症级联反应、氧化应激增强和BBB完整性破坏。总体而言，放疗所致神经损伤与总剂量、单次分割剂量、受照体积、正常组织剂量-体积参数及既往治疗暴露密切关联，具有较明确的剂量生物学基础。

放疗相关CNS毒性以放射性脊髓病和认知功能损害最具临床意义，二者均具有一定迟发性及潜在不可逆性，是放疗计划制定和随访管理中的重点。首先，放射性脊髓病是胸部放疗尤其脊柱或椎旁病灶高剂量照射时需高度警惕的严重并发症。其主要机制包括少突胶质细胞损伤导致的脱髓鞘改变、轴突传导障碍、微血管内皮损伤及局部缺血坏死等^[25]^。临床上可表现为感觉异常、肢体无力、步态障碍，严重者可进展为截瘫或括约肌功能障碍。既往研究^[26]^表明，脊髓SRT后放射性脊髓病发生率为0.3%-1.0%，虽然总体发生率较低，但一旦发生往往难以逆转。因此，在胸椎旁或椎体转移灶行放疗时，应重视对脊髓最大受照剂量、受照长度及既往再程照射累积剂量的综合评估。其次，放疗相关认知功能障碍是脑转移患者接受WBRT后最具代表性的中枢神经毒性之一。其病理机制较为复杂，主要涉及海马区神经发生抑制、突触可塑性下降、神经胶质细胞活化、白质微结构损伤以及持续性神经炎症^[27]^。与局灶性放疗相比，WBRT因受照范围广，对与学习记忆和执行功能密切相关的海马及额顶叶网络影响更为显著。临床研究^[27]^显示，接受WBRT的患者中30%-50%的患者在治疗后6个月内出现不同程度认知功能下降，以近期记忆、注意力和执行功能受损最为常见。值得注意的是，NRG Oncology CC001研究^[28]^证实，在美金刚辅助治疗基础上采用海马回避WBRT，可在不降低颅内控制率的前提下，显著降低认知功能衰退风险，降幅约为26%，提示保护关键功能脑区的精准放疗理念具有明确临床价值。因此，对于预计生存期较长、基线认知功能较好或神经认知保留价值较高的患者，海马回避策略应优先纳入技术可行性评估。

相较于CNS损伤，放疗相关PNS损伤多与照射野邻近的重要神经干、神经丛或自主神经结构受累有关，其发生机制以慢性纤维化、微血管闭塞和继发性神经缺血为主，临床上更易表现为慢性、隐匿进展性功能障碍。放射性神经丛病是典型代表，多见于臂丛神经或腰骶神经丛受照后，表现为慢性疼痛、麻木、感觉异常、肌力下降，部分患者可伴进行性运动功能障碍。其病理基础主要为慢性辐射损伤后局部纤维化、神经束周围瘢痕形成、微血管灌注减少及继发性脱髓鞘改变。对于肺尖部肿瘤、锁骨上区转移淋巴结或纵隔上部病灶放疗患者，臂丛神经剂量限制尤为重要^[29]^。此外，胸部及纵隔照射还可能累及喉返神经、迷走神经及膈神经等重要周围神经和自主神经结构。喉返神经受损可表现为声音嘶哑、咳嗽无力及误吸风险增加；迷走神经受累可引发吞咽功能障碍、胃肠动力异常或心率调节异常；膈神经损伤则可能导致膈肌运动减弱及呼吸困难。尽管此类并发症总体发生率低于中枢放疗毒性，但其对生活质量和呼吸循环功能的影响不容忽视，尤其在基础肺功能储备较差患者中更具临床后果。

放疗相关神经毒性的防控核心在于精准勾画、严格限量和动态评估。一方面，应基于剂量-体积约束原则，对脊髓、脑干、海马、视神经通路及神经丛等危及器官进行精细勾画，并结合既往照射史进行累积剂量评估；另一方面，应充分利用图像引导放疗、容积旋转调强放疗、脑转移同步加量及海马回避WBRT等技术，在维持局部控制率的同时最大限度减少正常神经组织受照^[30,31]^。此外，治疗前后应进行系统的神经功能动态评估，包括肌力、感觉、吞咽、发声及神经认知量表等，以提高早期识别率并指导分层干预。

#### 2.1.2 介入治疗

近年来，介入治疗作为肺癌局部治疗的重要补充手段发展迅速，主要包括支气管动脉栓塞化疗（bronchial arterial chemoembolization, BACE）、射频消融、微波消融、冷冻消融及放射性粒子植入等。该类技术具有微创、可重复实施及全身毒性相对较低等优势，特别适用于不能耐受手术、局部复发或寡转移病灶的患者。总体上，介入治疗相关神经系统并发症发生率低于广泛照射或开放手术，但在特定解剖部位、特殊能量模式或血管变异条件下，仍可造成具有实质临床后果的神经损伤。

介入治疗所致CNS损伤相对少见，但一旦发生往往起病急骤、后果严重。其中，BACE相关脊髓缺血性损伤是最值得警惕的严重并发症之一。由于支气管动脉、肋间动脉及脊髓供血血管之间存在复杂侧支交通，若栓塞材料误入脊髓前动脉或相关分支，可导致急性脊髓灌注障碍，临床表现为突发性下肢无力、感觉平面改变甚至截瘫^[32]^。该类损伤的本质是血管性缺血坏死，而非单纯机械刺激，因此其预后较大程度上取决于缺血范围及再灌注时机。基于此，术前精细化血管解剖评估、数字减影血管造影识别危险侧支以及超选择性插管，是预防此类严重神经并发症的关键措施。此外，放射性粒子植入虽然以低剂量率持续照射为特点，通常具有较好的局部适形性，但若粒子分布不均、位置偏移或与重要神经结构距离过近，仍可能形成局部热点效应并诱发迟发性中枢神经损伤。其机制主要涉及持续DNA双链断裂、神经胶质细胞凋亡及微环境炎症改变^[33]^。由于其临床表现可具有潜伏期，故粒子植入后不应仅关注局部控瘤效果，还需结合影像和神经功能随访进行综合评估。

介入治疗相关PNS损伤多由物理能量扩散或低温损害所致，其发生具有显著的解剖邻近性。热消融治疗，如射频消融和微波消融，主要通过高温诱导肿瘤细胞蛋白变性和凝固性坏死，但热传导及热辐射的旁及效应可损伤病灶邻近神经结构。肺尖部肿瘤消融时，臂丛神经因解剖位置邻近而存在受热损伤风险，临床可表现为患侧上肢麻木、疼痛、感觉减退或肌力下降；纵隔旁病灶消融则可能波及迷走神经，严重时可出现心律失常、胃肠功能紊乱等自主神经症状^[34]^。这提示介入治疗的安全边界不仅取决于肿瘤大小和消融范围，还高度依赖邻近关键神经结构的空间关系。与热消融不同，冷冻消融通过细胞内外冰晶形成、渗透压变化及微循环灌注阻断诱导肿瘤细胞死亡。神经组织对低温亦较敏感，但其损伤在一定温度和时程内可表现为以髓鞘改变为主的可逆性传导阻滞，而非完全性轴索断裂^[35]^。因此，冷冻消融所致PNS损伤多具有一定恢复潜能，术后经支持治疗和随访观察后，多数患者神经功能可逐渐改善。相较于不可逆热坏死，冷冻消融在靠近某些重要神经结构时可能具备一定安全优势，但仍需严格控制冰球边界和暴露时长。

介入治疗神经并发症的防控关键在于术前路径设计、术中实时监测及术后功能评估的全流程优化。对于热消融和冷冻消融，三维影像导航、实时温度监测及邻近重要结构的安全边界预判可有效降低神经损伤风险；对于BACE，血管变异识别和超选择性栓塞是避免脊髓缺血的核心；对于放射性粒子植入，则应强化精准剂量学规划、粒子空间分布优化及术后位置复核，以减少迟发性辐射性神经损伤。

#### 2.1.3 外科治疗

外科切除仍是可切除肺癌的核心局部治疗方式，在早期肺癌根治及部分脑转移灶减瘤治疗中具有重要地位。与放疗和介入治疗相比，外科治疗相关神经损伤更强调直接解剖损害的特点，其主要机制包括术中牵拉、压迫、电凝热损伤、血供破坏及神经离断，损伤形式更为直接，临床表现亦更具器官定位特征。

外科治疗对CNS的影响主要见于肺癌脑转移瘤切除术。由于肿瘤切除过程中不可避免涉及瘤周脑组织、局部血管及功能区通路操作，患者术后可出现癫痫发作、肢体运动功能障碍、语言障碍或认知功能受损等神经系统并发症。有报道^[36]^显示5%-15%的患者在术后出现短暂性神经功能缺损。其机制不仅包括手术操作所致的直接脑组织损伤，还涉及局部BBB破坏、术后炎症反应、脑水肿及微循环障碍等一系列继发性病理过程。对此类患者而言，术前功能区评估、术中神经导航及围手术期脑水肿管理对于降低中枢神经并发症至关重要。

与CNS损伤相比，肺癌外科治疗中PNS损伤更为常见，且多与胸腔解剖毗邻结构有关。首先，肋间神经损伤是胸外科术后最常见的PNS损伤并发症之一，可由切口牵拉、肋间撑开、器械压迫及局部瘢痕形成所致，进而诱发术后疼痛综合征^[37]^。该综合征常表现为持续性胸壁疼痛、感觉异常或触诱发痛，不仅影响患者术后活动和肺康复依从性，还可能演变为慢性神经病理性疼痛，进而损害长期生活质量。其次，中央型肺癌或纵隔淋巴结清扫手术中，喉返神经因紧邻纵隔及肺门结构而易受损。其临床表现主要为声音嘶哑、吞咽困难、呛咳及误吸风险增加；单侧损伤较为常见，而双侧损伤则可能因声带麻痹引发严重上气道梗阻，甚至危及生命^[38]^。因此，对于肺门、隆突周围及上纵隔操作范围较大的患者，术中识别和保护喉返神经尤为关键。此外，交感神经链损伤虽相对少见，但在上胸段病灶切除或纵隔操作时仍可能发生，典型表现为Horner综合征，即同侧上睑下垂、瞳孔缩小及面部无汗^[39]^。这类并发症虽通常不直接危及生命，但提示术中神经保护不足，也可能对患者术后功能和感知造成持续影响。

随着微创技术的发展，电视辅助胸腔镜手术及达芬奇机器人辅助手术在肺癌外科中的应用日益广泛。与传统开胸手术相比，微创技术通过缩小切口、减少肋间撑开程度、提升术野放大效果和操作精细度，在降低术中失血和术后疼痛的同时，也在一定程度上减少了神经损伤发生率^[40]^。然而，微创并不意味着无神经风险，尤其在肿瘤侵犯范围广、局部解剖紊乱或需扩大切除的情况下，神经并发症仍难以完全避免。因此，外科治疗中神经保护的关键在于：术前基于影像学准确评估肿瘤与重要神经结构关系，术中实施标准化解剖与轻柔操作，术后联合疼痛管理和神经功能康复，以实现控瘤效果与功能保留的平衡。

### 2.2 全身治疗

#### 2.2.1 化疗

化疗在肺癌综合治疗体系中仍占据核心地位，对晚期无驱动基因突变的NSCLC及SCLC患者具有不可替代的价值。然而，传统以铂类和紫杉烷类为代表的化疗药物缺乏肿瘤特异性，在杀伤肿瘤细胞的同时亦可损伤正常组织，导致多系统毒性反应。其中，化疗相关周围神经病变（chemotherapy-induced peripheral neuropathy, CIPN）是最常见且影响治疗依从性和生活质量的神经系统并发症之一^[41]^。多中心研究^[42]^显示，接受紫杉烷类或铂类治疗的肺癌患者中，CIPN总体发生率为40%-68%，其中20%-30%为≥2级神经毒性，约15%的患者需因此进行剂量调整或延迟治疗。

CIPN的主要临床表现为以感觉神经损伤为主的周围神经病变，患者多出现肢端麻木、刺痛及痛觉过敏等症状；病情进展时可累及运动神经，引起肌无力、痉挛及平衡障碍等，尤以接受紫杉烷类药物治疗者更为明显^[43]^。CIPN的发生主要与化疗药物种类、累积剂量及治疗暴露有关。目前尚无充分证据表明肺癌脑转移患者的CIPN发生率低于无脑转移患者。尽管脑转移患者在临床上可能更多接受局部治疗及具有中枢活性的系统治疗，从而减少神经毒性化疗暴露，但该因素是否导致CIPN风险下降仍有待进一步研究证实^[44]^。

在分子机制层面，不同化疗药物通过多重途径损伤PNS。紫杉烷类药物主要通过稳定微管结构、抑制微管动态重组，造成微管动力学失衡，进而干扰轴突内物质运输过程；长春花生物碱类则通过抑制微管聚合，破坏轴突运输的结构基础，进一步影响神经传导功能；铂类化合物主要累及感觉神经元，通过诱导背根神经节神经元DNA损伤与细胞凋亡，同时伴随髓鞘结构破坏及神经炎症反应激活^[45]^。其共同结果为以轴突变性为核心的神经退行性病变，表现为神经传导减慢及功能障碍加重。髓鞘破坏进一步阻断神经冲动正常传递，二者协同形成CIPN的主要病理生理基础^[46]^。此外，炎症因子介导的免疫反应在CIPN发生发展中同样发挥重要作用。研究显示，IL-6、肿瘤坏死因子-α（tumor necrosis factor-α, TNF-α）等促炎介质可激活MAPK及核因子-κB（nuclear factor-κB, NF-κB）信号通路，诱导神经元氧化应激和细胞凋亡，从而加剧神经损伤和疼痛敏化^[47]^。进一步研究^[48]^表明，施万细胞（Schwann cells, SCs）可通过分泌半乳糖凝集素-3（galectin-3）促进巨噬细胞向紫杉烷诱导的CIPN部位浸润，从而放大神经炎症反应并加重神经病理性疼痛。

针对CIPN的干预以剂量调整和对症治疗为主，其中度洛西汀目前具有较为明确的循证医学证据，加巴喷丁及普瑞巴林亦可用于缓解神经性疼痛，抗氧化剂等辅助治疗手段尚缺乏一致性证据支持。除CIPN外，接受化疗的肺癌患者亦存在认知功能障碍风险升高，统称为癌症相关认知障碍。其发生与化疗药物诱导的脑结构重塑及神经功能网络异常密切关联。相较于PNS损伤，化疗对CNS的影响机制尚未完全阐明。因此，未来研究应系统探讨化疗相关中枢神经毒性的分子基础与神经调控途径，为临床制定综合性神经毒性防治策略提供理论支撑与实践依据。

#### 2.2.2 免疫治疗

免疫检查点抑制剂（immune checkpoint inhibitors, ICIs）作为单药或联合化疗方案，已成为转移性肺癌的标准一线治疗选择，尤其在缺乏可靶向驱动基因突变的患者中占据核心地位。随着ICIs在临床中的广泛应用，其免疫相关不良反应（immune-related adverse events, irAEs）逐渐受到关注，其中神经系统毒性虽总体发生率较低，但临床后果可能较为严重。现有研究^[49]^显示，ICIs相关神经系统irAEs总体发生率为1%-5%，其中≥3级严重神经毒性发生率为0.5%-1%。在合并副肿瘤性神经系统综合征（paraneoplastic neurological syndromes, PNSs）的患者中，约40%需永久停用免疫治疗，且部分患者存在神经功能恢复不完全的临床结局。回顾性分析^[50]^显示，发生严重神经irAEs的患者其中位无进展生存期较未发生者缩短3-5个月，提示神经毒性可能通过影响治疗持续性而间接降低患者获益。

在临床表现方面，ICIs相关神经毒性具有高度异质性，可累及CNS及PNS。常见表现包括重症肌无力、Lambert-Eaton肌无力综合征、副肿瘤性边缘叶脑炎、小脑变性及感觉神经病变等^[51]^，多数可归属于PNSs范畴。上述疾病可表现为肌无力、共济失调、认知功能障碍及感觉异常等不同临床谱系，部分患者病情进展迅速，甚至可危及生命。

在分子机制层面，PNSs的主要致病机制与肿瘤神经抗体的异常产生密切关联，该类抗体来源于机体对肿瘤细胞抗原的异常适应性免疫反应。其发病核心在于肿瘤神经抗体可与正常神经元表面或胞内抗原发生特异性结合，诱发交叉免疫反应，进而引起神经系统的炎症性损伤与结构性破坏，导致进行性或不可逆的神经功能障碍^[52]^。肿瘤细胞在凋亡或坏死过程中释放细胞表面抗原或细胞内抗原后，被树突状细胞摄取，经抗原加工后在区域淋巴结中通过主要组织相容性复合体（major histocompatibility complex, MHC）分子提呈给CD4^+^或CD8^+^ T细胞，启动特异性免疫反应^[53]^。在此过程中，树突状细胞经MHC II类分子提呈抗原激活CD4^+^ T细胞，后者与B细胞相互作用，诱导其分化为浆细胞和记忆B细胞。这些活化的B细胞可穿越BBB，在CNS局部微环境中进一步激活，分泌大量自身抗体。抗体与脑内神经元膜表面抗原结合后，可通过补体依赖性细胞毒作用或抗体依赖性细胞介导的细胞毒性途径介导CNS的损伤，从而造成神经元功能障碍与网络异常。相较之下，CD8^+^ T细胞经MHC I类分子提呈抗原后被活化，主要识别并攻击表达胞内抗原的神经元，发挥细胞毒性作用，是PNSs另一关键的免疫致病通路。若靶抗原定位于PNS，自身抗体可与神经肌肉接头突触前膜的电压门控钙通道结合，抑制神经递质释放，导致神经肌肉传导阻滞，从而出现以肌无力为特征的临床表现^[54]^。

在干预策略方面，ICIs相关神经毒性管理的核心在于早期识别与及时免疫抑制。对于≥2级神经毒性通常建议暂停免疫治疗并启动糖皮质激素治疗；对于≥3级或进展迅速的严重病例，多需永久停用ICIs并给予大剂量激素治疗。对于激素反应不佳或危重患者，可进一步考虑静脉注射免疫球蛋白或血浆置换等免疫调节手段。建立针对高风险人群如SCLC患者或肿瘤神经抗体阳性个体的动态监测与早期干预机制，对于降低神经毒性相关不良结局具有重要临床意义。

#### 2.2.3 靶向治疗

靶向治疗相关神经毒性具有明显的药物特异性，其发生风险与药物的CNS穿透能力及治疗暴露有关。在驱动基因阳性的NSCLC中，EGFR突变及间变性淋巴瘤激酶（anaplastic lymphoma kinase, ALK）基因重排可激活多条下游信号通路，显著增强肿瘤细胞侵袭及转移潜能，其中脑转移是导致患者死亡的重要原因之一^[55]^。受BBB通透性限制，第一代EGFR-TKIs（如吉非替尼、厄洛替尼）及第一代ALK-TKIs（克唑替尼）对脑转移的控制能力相对有限；第二代EGFR-TKIs 的中枢活性较第一代有所改善，但总体仍逊于第三代EGFR-TKIs。相比之下，第二代ALK-TKIs（如阿来替尼、布格替尼）以及以洛拉替尼为代表的新一代ALK-TKIs具有更好的CNS渗透能力和颅内控制活性，已成为ALK阳性NSCLC合并脑转移患者的重要一线治疗选择。第三代EGFR-TKIs则在EGFR突变阳性NSCLC脑转移患者中显示出更优的中枢控制获益。III期CROWN研究^[56]^结果显示，第三代ALK-TKIs洛拉替尼可显著降低脑转移发生风险。III期ADAURA研究^[57]^系统评估了奥希替尼在完全切除的IB-IIIA期EGFR突变阳性NSCLC患者中的术后辅助治疗价值，结果表明该药物不仅显著改善无病生存期，同时明显降低包括CNS在内的远处复发风险，CNS获益主要体现在复发预防。与此不同，FLAURA研究^[58]^在IV期EGFR突变阳性NSCLC一线治疗人群中证实奥希替尼具有更优的CNS控制能力，从而在转移性疾病中确立其一线治疗地位。尽管靶向药物在CNS转移的防治中展现出显著疗效，能够有效降低脑转移风险，但其亦可能引发不同程度的神经系统AEs。真实世界数据分析^[59]^显示，出现≥2级CNS不良事件的患者中约28%需剂量调整，约8%暂时中断治疗；神经认知不良事件与既往脑转移及既往放疗史有显著关系[比值比（odds ratio, OR）=2.14，95%CI: 1.32-3.47]。

在临床表现方面，靶向治疗相关神经毒性主要以CNS症状为主，表现为头晕、癫痫发作、认知功能障碍、情绪及行为改变以及共济失调等。其中，第三代ALK-TKIs洛拉替尼最具代表性。在CROWN研究^[56]^中洛拉替尼组神经系统不良事件总体发生率达35%，中位发生时间为57 d，中位持续时间为182 d；约62%的患者无需特殊干预即可改善，仅约2%的患者因AEs需永久停药。此外，神经营养TKIs如恩曲替尼及拉罗替尼亦可引发多种神经系统症状，包括头晕、共济失调、直立性低血压及外周感觉神经病变等。值得注意的是，靶向治疗相关神经毒性多为轻中度，且具有一定可逆性，但在部分患者中仍可能影响生活质量及治疗依从性^[60]^。

在分子机制层面，靶向治疗相关神经毒性主要与药物在CNS中的暴露增加及其对神经信号通路的干扰有关。驱动基因阳性NSCLC细胞与神经系统之间存在复杂的双向调控关系：一方面，EGFR突变肿瘤细胞可诱导小胶质细胞分泌IL-6、IL-8及TNF-α等促炎因子，激活神经炎症反应并破坏BBB，从而促进肿瘤细胞在脑内定植^[61]^；另一方面，脑微环境亦可通过脑微血管相关肿瘤生态位增强肿瘤细胞适应性并促进耐药性形成^[62]^。此外，星形胶质细胞可通过上调代谢型谷氨酸受体1（metabotropic glutamate receptor 1, mGluR1），使EGFR阳性肿瘤细胞对谷氨酸信号产生依赖，进一步促进其在脑内生长与扩增^[63]^。与此同时，ALK等靶点在神经系统中的表达亦提示靶向药物可能直接干扰神经细胞功能，从而诱发CNS相关AEs。

在干预策略方面，靶向治疗相关神经毒性的管理强调风险收益平衡及个体化剂量调控。对于轻度症状患者，可在严密监测下继续治疗；当出现≥2级AEs时，通常建议优先进行剂量下调或短期停药；对于持续或加重的症状，应综合评估肿瘤控制情况及神经毒性恢复程度后决定是否调整治疗方案。值得注意的是，多数患者的神经系统AEs在剂量调整或支持治疗后可得到改善，仅少数患者需永久停药。建立基于基线神经功能评估及动态监测的管理策略，对于降低神经毒性风险并维持治疗连续性具有重要意义。不同肺癌治疗策略对神经系统的双重影响及作用机制比较见表1。

**表1:** 不同肺癌治疗策略对神经系统的双重影响及作用机制比较

Treatment strategy	Representative drugs/techniques	Anti-tumor neuro-related effects	Adverse neurological effects	Key mechanisms
Local treatment	Surgery, IMRT, SRS, WBRT, ablation therapy, etc.	Control of brain metastasis, alleviation of space-occupying symptoms; precise radiotherapy reduces exposure of normal brain tissue	Radiation myelitis, radiation-induced brain damage, cognitive decline, post-operative nerve damage (recurrent laryngeal nerve, sympathetic chain), peripheral nerve damage	Ionizing radiation damages neurons and microvascular endothelial cells; demyelination; suppression of hippocampal neurogenesis; intraoperative direct nerve damage or ischemia
Chemotherapy	Platinum agents, Taxanes, Vincristine, etc.	Systemic anti-tumor effects; certain regimens reduce brain metastasis risk	CIPN; CRCI	Disruption of microtubule dynamics leads to axonal transport impairment; DRG neuronal apoptosis; myelin sheath damage; IL-6/TNF-α-mediated neuroinflammation; oxidative stress and mitochondrial damage
Immunotherapy	PD-1/PD-L1 inhibitors, etc.	Improved survival in advanced patients; enhanced anti-tumor immunity	PNSs; myasthenia gravis, paraneoplastic encephalitis, cerebellar degeneration, sensory neuropathy	Tumor neuroantibody generation; abnormal activation of CD4^+^/CD8^+^ T cells; BBB disruption; complement- and ADCC-mediated neurotoxic effects
Targeted therapy	EGFR-TKIs, ALK-TKIs, etc.	Good CNS penetration; reduced brain metastasis occurrence; improved CNS control	Dizziness, seizures, cognitive impairment, mood changes, ataxia, sensory neuropathy	High BBB permeability leads to increased CNS exposure; interference with neural signaling pathways; glial cell-tumor interaction alters microenvironment

IMRT: intensity modulated radiation therapy; SRS: stereotactic radiosurgery; WBRT: whole brain radiotherapy; CIPN: chemotherapy-induced peripheral neuropathy; CRCI: cancer-related cognitive impairment; DRG: dorsal root ganglion; IL-6: interleukin-6; TNF-α: tumor necrosis factor-α; PD-1: programmed cell death protein 1; PD-L1: programmed cell death ligand 1; PNSs: paraneoplastic neurological syndromes; BBB: blood-brain barrier; ADCC: antibody-dependent cell-mediated cytotoxicity; EGFR-TKIs: epidermal growth factor receptor-tyrosine kinase inhibitors; ALK: anaplastic lymphoma kinase; CNS: central nervous system.

## 3 肺癌药物治疗相关神经系统AEs的处理

在前述不同治疗方式神经毒性特征基础上，本部分进一步从临床管理角度进行系统归纳。在启动化疗、免疫治疗或具有较高CNS穿透能力的靶向治疗前，应开展系统化的神经系统基线评估，以实现治疗相关神经毒性的前瞻性风险分层与动态管理。评估内容应包括标准化神经系统体格检查、认知功能量表测评、既往神经系统疾病史及脑放疗史的系统回顾、影像学对脑转移负荷的客观评估，以及精神心理共病状态筛查。建议采用美国国家癌症研究所不良事件通用术语标准（National Cancer Institute-Common Terminology Criteria for Adverse Events, NCI-CTCAEs）对神经系统症状进行分级与记录，以建立可纵向比较的监测基线，为治疗过程中毒性识别及干预决策提供量化依据^[65]^。

对于化疗相关神经毒性，其代表性表现为CIPN，具有明确的剂量累积效应和以周围感觉神经损伤为主的特征。其管理重点在于早期识别与暴露控制。在治疗过程中应通过动态评估及时发现神经毒性进展，并根据严重程度采取剂量调整、延迟给药或更换方案等措施，以防止不可逆神经损伤。对症治疗方面，神经调节类药物如度洛西汀、普瑞巴林及加巴喷丁可通过调控5-羟色胺、去甲肾上腺素及γ-氨基丁酸相关通路降低神经元异常兴奋性，从而缓解神经病理性疼痛，其中度洛西汀具有相对明确的循证医学证据支持。抗氧化剂如乙酰左旋肉碱及α-硫辛酸可通过减轻氧化应激和改善线粒体功能发挥一定神经保护作用，但其临床获益仍需进一步验证^[65]^。此外，机制导向干预逐渐受到关注，例如地氯雷他定通过调控5-HT2A/c-Fos/NLRP3信号通路抑制神经炎症反应，为CIPN的精准干预提供了新的研究方向^[66]^。

对于irAEs，其本质为免疫介导性炎症损伤，虽发生率较低，但进展迅速且可能造成严重甚至不可逆后果。因此，其管理策略不同于化疗，更强调早期识别与快速免疫抑制。临床上应遵循分级干预原则：1级毒性以密切监测为主；2级毒性通常需暂停免疫治疗并启动糖皮质激素治疗；对于≥3级严重神经毒性，应永久停用免疫治疗并给予大剂量糖皮质激素，必要时联合静脉注射免疫球蛋白或血浆置换等免疫调节措施。尤其在PNSs背景下，早期诊断和及时干预对于防止神经功能不可逆损害具有决定性意义^[67]^。因此，免疫治疗相关神经毒性的处理关键在于降低干预阈值并尽早启动系统性免疫调控。

对于靶向治疗相关神经毒性，其临床表现与药物CNS穿透能力及靶点生物学特性密切关联。以第三代ALK-TKIs洛拉替尼为代表，其神经认知异常具有一定剂量依赖性且多为可逆性改变。相较于免疫治疗需迅速停药的策略，靶向治疗更强调剂量调整与风险收益平衡。对于轻度中枢神经症状，可在严密监测下继续治疗；当出现≥2级毒性时，建议优先进行剂量下调；若症状持续或加重，则需暂时停药，并结合肿瘤控制情况及神经毒性恢复程度综合评估后决定后续治疗策略^[68]^。该管理模式体现了在保证CNS控瘤获益的同时，最大程度维持治疗连续性的原则。

综上，肺癌药物治疗相关神经系统AEs的处理应在统一的风险评估与分级管理框架下，实施基于治疗类型的差异化干预策略：化疗以剂量管理与症状控制为核心，免疫治疗以快速免疫抑制为关键，靶向治疗则以剂量调控与风险收益平衡为重点，有助于在保障抗肿瘤疗效的同时最大限度降低神经系统损伤，提高患者长期生存质量。针对肺癌相关自身抗体介导的特殊神经系统损伤，免疫调节治疗仍是临床控制的核心策略，必要时可联合抗精神病药物或苯二氮卓类药物实施症状性管理。总体而言，深入解析肺癌细胞与神经系统之间复杂的相互作用网络，阐明肿瘤微环境中关键神经调控信号通路及其分子机制，并据此开发靶向干预策略，对于减少肺癌相关神经系统损伤、优化治疗效果及改善患者预后具有重要研究价值与临床意义。

## 4 小结

神经系统在肺癌的发生、进展及治疗反应中发挥多维调控作用，其通过神经支配、神经递质释放、神经营养因子信号及神经-免疫互作等机制，参与肿瘤细胞增殖、侵袭、转移及治疗耐受等关键过程。同时，肺癌及相关治疗干预可反向影响神经系统的结构与功能稳态，导致放射性神经损伤、CIPN、免疫相关神经毒性及靶向治疗相关CNS AEs，从而对患者生活质量与治疗依从性产生重要影响。

基于现有证据，本课题组认为，肺癌的神经调控网络不仅是肿瘤生物学研究的重要基础，也是精准治疗优化的重要切入点。未来研究应聚焦以下方向：（1）系统解析肺癌微环境中神经-免疫-肿瘤互作的关键信号通路，明确其在肿瘤侵袭、转移及免疫耐受中的作用机制；（2）开发靶向神经调控的干预策略，包括基于自主神经或中枢神经调控的精准干预，以改善治疗相关神经毒性及提升疗效；（3）利用AI、多组学及数字化神经影像等技术，实现神经毒性风险的前瞻性预测和个体化管理。总体来看，通过加强神经科学与肿瘤学的协同研究，深入解析肺癌神经调控机制并推动神经调控策略的临床转化，有望为肺癌精准防治提供新的理论基础与干预路径。
